# Frequency of Various Foreign Bodies Retrieved from the Airway During Bronchoscopy in Children: A Pediatric Tertiary Care Center Experience

**DOI:** 10.7759/cureus.9348

**Published:** 2020-07-22

**Authors:** Rewa Chand, Mehmood Shaikh, Yousuf Khan, Mumtaz Ahmed Qureshi, Hemandas Maheshwari, Mehrunnisa Yasir

**Affiliations:** 1 Pediatric Surgery, Shaheed Mohtarma Benazir Bhutto Medical College, Karachi, PAK; 2 Neonatal Intensive Care Unit, Jinnah Sindh Medical University, Karachi, PAK; 3 Pediatric Surgery, Abbottabad International Medical College, Abbottabad, PAK; 4 Pediatric Surgery, Liaquat University of Medical & Health Sciences, Hyderabad, PAK; 5 Pediatrics, St. Richard's Hospital, Chichester, GBR; 6 Medical Intensive Care Unit, National Institute of Child Health, Karachi, PAK

**Keywords:** foreign body aspiration, tracheobronchial, betel nut, rigid bronchoscopy, children

## Abstract

Objective: To determine the frequency of various foreign bodies (FBs) retrieved from the airway during bronchoscopy in children at the National Institute of Child Health (NICH), Karachi, Pakistan.

Study Design: Cross-sectional descriptive study.

Place and Duration: Department of Pediatrics Surgery, NICH, Karachi, Pakistan from June 1, 2017 to November 30, 2017.

Methodology: Patients referred from the ER and Pediatrics Medicine Department, NICH, Karachi with a suspicion of tracheobronchial foreign body aspiration (FBA) was included in the study.

Results: A total of 96 children were studied. There were 71 males (74%) and 25 females (26%). Eighty-seven (90.6%) were below five years and nine (9.4%) were more than five years of age. Mean time interval between FBA and presentation at hospital was 15 h. FB was located primarily in the right main bronchus (54%), followed by left bronchus (40%) and trachea (6%). Betel nut was the most common FB retrieved in 87.5%. Other FBs were whistle 3.1%, peanut 3.1%, seed 1%, and miscellaneous 5.2%.

Conclusions: FBA is more common in male children, mostly below five years of age. During bronchoscopy, it was found that the FB was mostly located in the right main bronchus. Betel nut was found to be the most common FB aspirated.

## Introduction

Foreign body aspiration (FBA) in pediatric population remains a life-threatening condition [1]. Around the globe, eight children die every hour due to FBA, and pre-school children are the most affected [2-3]. It is the fifth common cause of unintentional death between one- and two-year-old children and the principal cause of accidental death under one year [4-5].

The FBA mostly involves one to three years age group children due to their habit of taking small objects in mouth because of their explorative nature in order to determine their taste and texture and chewing while teething [6-7]. Foreign bodies (FBs) of organic origin such as peanuts, seeds, and food particles are commonly attracted and aspirated in young children while nonorganic ones e.g. coins, paper clips, pins, and pen caps are more usually aspirated by older children [8].

Aspirated FB is mostly impacted in the bronchi, less commonly in the trachea and larynx and, this impaction site depends upon its size [9]. It commonly manifests as sudden onset choking, wheezing, diminished breath sounds, and unilateral crackles [10]. Delayed diagnosis can lead to deterioration of the patient’s clinical condition and may result in serious complications such as bronchitis, tracheitis, atelectasis, and pneumonia. Early diagnosis and management are important to minimize these severe complications [11-12]. Rigid bronchoscopy is a diagnostic as well as therapeutic modality in suspected cases of FBA [4].

The diagnosis of FBA is based on detailed history and physical examination, abnormal radiological findings, and eventual removal of the aspirated FB by rigid bronchoscopy [13]. About 80% of swallowed FB passes through the gastrointestinal tract spontaneously while remaining 20% require intervention. Only less than 1% of ingested FB cases require surgical intervention either for retrieval or for the management of complications [14]. The aim of the study is to share personal experiences of rigid bronchoscopy as a diagnostic and therapeutic tool in children with suspected FBA at a pediatrics tertiary care center.

## Materials and methods

This was a cross-sectional descriptive study conducted at the Pediatric Surgery Department, National Institute of Child Health (NICH), Karachi, for a period of six months, from June 1, 2017 to November 30, 2017.

The study was initiated after approval from the Institutional Review Board and after obtaining informed consent from parents/guardians. Children between 1 and 12 years of age presenting with a history of FBA within 24 h having one or more of the following signs: choking, temperature >100^o^F, cough, and wheeze on auscultation after inhalation of FB were labeled as FBA and were included in the study by consecutive nonprobability sampling. Patients more than 12 years, presenting >24 h or bronchoscopy performed for diagnostic purposes (bronchial lavage or biopsy) were excluded from the study. The data including history, examination, time interval between clinical presentation and diagnosis, and type and location of FB were collected on specially designed proforma. A chest X-ray was done and rigid bronchoscopy was done under general anesthesia by a pediatric surgeon. Results were analyzed using SPSS-20. Numerical variables are presented in the form of frequency and percentages.

## Results

**Figure 1:**
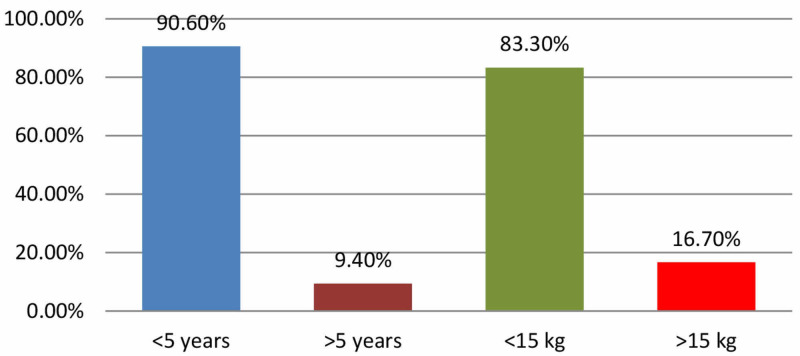
Age and weight of the patients with FBA. FBA, foreign body aspiration

**Figure 2:**
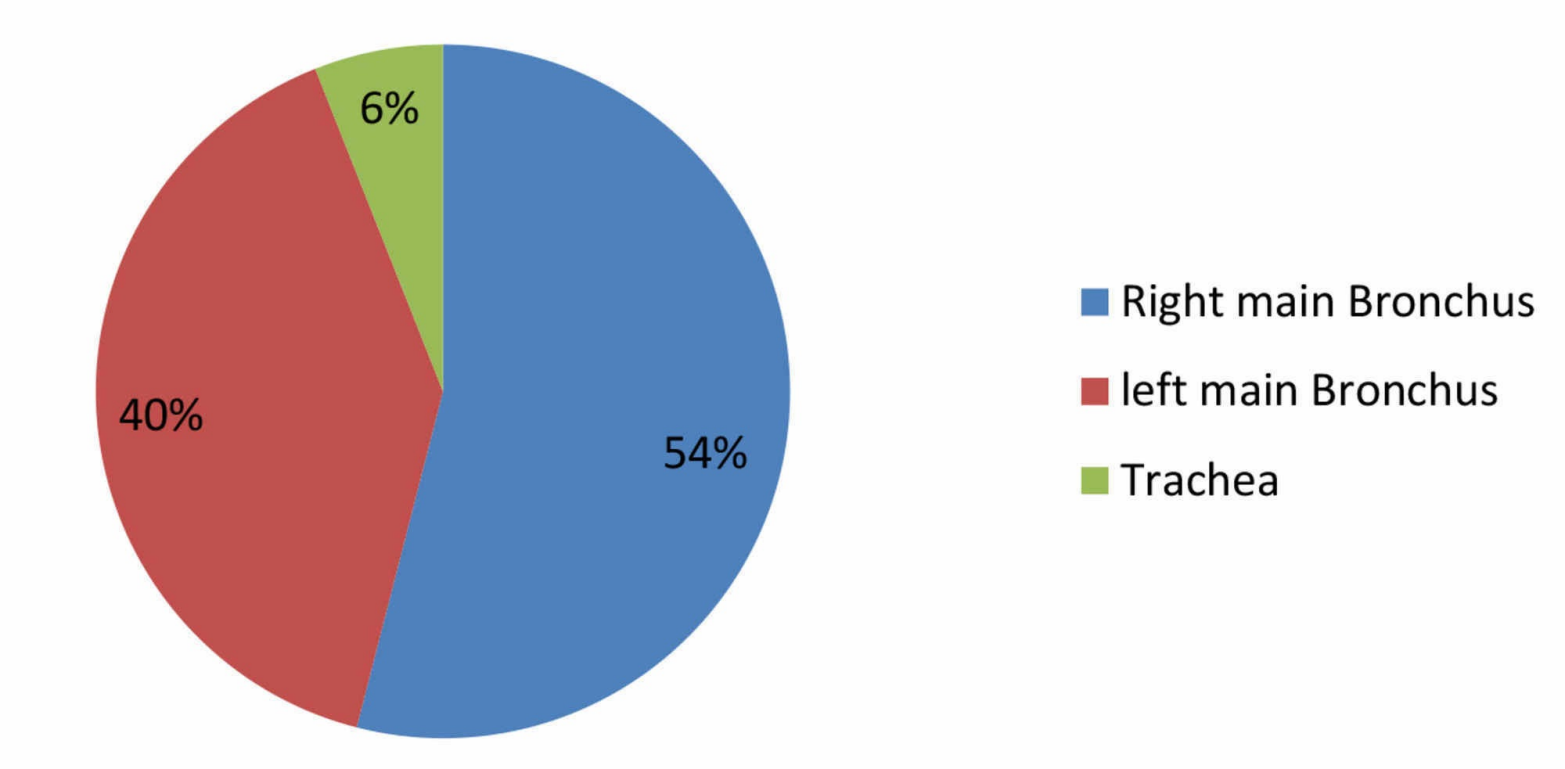
Location of the FB during bronchoscopy (N = 96). FB, foreign body

**Figure 3:**
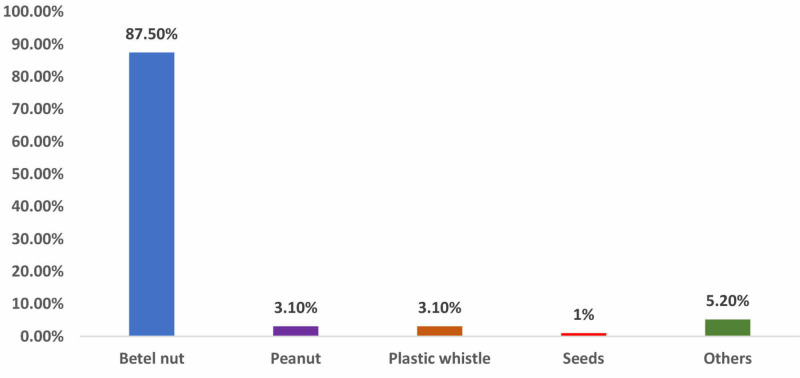
Various types of FBs retrieved from the airway during bronchoscopy (N = 96). FBs, foreign bodies

A total of 96 cases were included in the study. Out of 96 children 71(74%) were males and 25(26%) were females resulting in male: female ratio of 2.8:1. Among these, 87(90.6%) were under five years of age and 80(83.3%) children were <15 kg in weight (Figure 1). Mean time interval between FBA and presentation at hospital was 15 h. During bronchoscopy, FB was found located primarily in right main bronchus 52(54%), left bronchus 38(40%), and trachea 6(6%) (Figure 2). Betel nuts were found in 84(87.5%) while peanuts and plastic whistle were removed in 3(3.1%) each (Figure 3).

## Discussion

Foreign body aspiration is a potentially fatal condition and accounts for 40% of all accidental deaths under one year of age [15]. Site of impaction and size of FB are main factors to determine the outcome of FBA [16].

In our study, 74% of children undergoing bronchoscopy were male. Similar results were also observed in the previous studies that have indicated that this condition is more common among male as compared to female children [17-20]. The reason for male predominance remains unclear, however, some attribute it to the more adventurous and impulsive nature of the young boys [21]. In the present study, majority of FBA was seen in children below five years of age (90.6%) which is similar to that reported by Korlacki et al. and Asif et al. [6,16]. The similarity in age groups is due to the fact that pre-school age group is attributed as the most investigational period of life and hence as highest risk of aspirations and related complications.

Foreign bodies were lodged in right and left main bronchi in 54% and 40%, respectively. Similar findings were observed in the study by Halwai et al. and Karazanis et al. and inconsistent with the findings of Safari and Manesh and Ahmed and Shuiabu in which most objects were seen lodged in the left bronchus [17, 22-24]. In a study conducted by Janahi et al. in Qatar, 6.6% of FBs were found in trachea [5]. The results conformed with our study where FBs were found in trachea in 6% of the studied children. Position of the FB mainly depends on the age and physical activity of patients at the time of aspiration [25].

In countries where betel nut is consumed excessively, it is the chief culprit in our study, retrieved in 87%. Other FBs included peanuts (3.1%), plastic whistle (3.1%), seeds (1%), and others in 5.2% cases. Contrary to this, in other studies the most commonly aspirated FB was peanut [6, 24], while the most common FB retrieved from Middle Eastern girls are scarf pins [26]; Safari and Manesh have reported seeds as the most common FB [23]. This difference may be due to education, dietary habits, and socio-cultural features of communities [27].

The diagnosis and early bronchoscopy extraction of a FB in children is diagnostic as well as a therapeutic life-saving procedure and it also helps to prevent long-term complications [28-29]. The ideal time to perform bronchoscopy is within first 24 h of the incident for an optimal outcome [30]. Rigid bronchoscopy and FB retrieval is the standard treatment in cases of suspected aspiration [3]. At our institution all cases of FBA were managed with rigid bronchoscopy.

## Conclusions

Preventing aspiration of a FB is the ideal strategy. Therefore, it is necessary to teach parents and care-givers through mass media not to give any small objects like nuts, seeds, and others to unattended pre-school children which can be swallowed. Rigid bronchoscopy should be performed within 24 h in all children with suspected FBA. The most common FBs were betel nut, peanut, and plastic whistle.
